# Growing poplars for research with and without mycorrhizas

**DOI:** 10.3389/fpls.2013.00332

**Published:** 2013-08-27

**Authors:** Anna Müller, Katharina Volmer, Manika Mishra-Knyrim, Andrea Polle

**Affiliations:** Forest Botany and Tree Physiology, Büsgen-Institut, Georg-August Universität GöttingenGöttingen, Germany

**Keywords:** poplar, mycorrhiza, fungi, laboratory protocols, *in vitro*, plant growth, micropropagation

## Abstract

During the last decades the importance of the genus *Populus* increased because the poplar genome has been sequenced and molecular tools for basic research have become available. Poplar species occur in different habitats and harbor large genetic variation, which can be exploited for economic applications and for increasing our knowledge on the basic molecular mechanisms of the woody life style. Poplars are, therefore, employed to unravel the molecular mechanisms of wood formation, stress tolerance, tree nutrition and interaction with other organisms such as pathogens or mycorrhiza. The basis of these investigations is the reproducible production of homogeneous plant material. In this method paper we describe techniques and growth conditions for the *in vitro* propagation of different poplar species (*Populus* × *canescens*, *P. trichocarpa*, *P. tremula*, and *P. euphratica*) and ectomycorrhizal fungi (*Laccaria bicolor*, *Paxillus involutus*) as well as for their co-cultivation for ectomycorrhizal synthesis. Maintenance and plant preparation require different multiplication and rooting media. Growth systems to cultivate poplars under axenic conditions in agar and sand cultures with and without mycorrhizal fungi are described. Transfer of the plants from *in vitro* to *in situ* conditions is critical and hardening is important to prevent high mortality. Growth and vitality of the trees *in vitro* and outdoors with and without ectomycorrhizas are reported.

## Introduction

Among woody plant genera, the genus *Populus* is of increasing economic and scientific importance. The genus consists of six sections with about 30–35 poplar species world-wide (Eckenwalder, 1996). The species occur in riparian and forest ecosystems across the Northern Temperate Zone from the polar circle to 30° north latitude (Lubrano, 1992; Dickmann, 2001). The economic value and natural distribution of the genus *Populus* varies between the sections. Some species, e.g., *P*. *tremuloides* and *P*. *tremula* have extensive distribution ranges across whole North America and Eurasia, respectively, whereas others are confined to distinct regions (Dickmann, 2001). The genetic variability within the genus allows for the selection of genotypes that are adapted to different site conditions such as poor, degraded, or polluted soils (Lubrano, 1992; Doty, 2008; Chen and Polle, 2010). *Populus* species and hybrids are grown worldwide for plywood, lumber, paper, woody biomass, and bioenergy production (Polle and Douglas, 2010; Sannigrahi et al., 2010; Polle et al., 2013).

Because of their increasing importance for biomass production and the availability of molecular tools, poplars are studied in a vast range of research areas such as wood formation and wood properties (Plomion et al., 2001; Pilate et al., 2004; Janz et al., 2012), nutrition (Harvey and van den Driessche, 1997; Cooke and Weih, 2005; Lukac et al., 2010; Rennenberg et al., 2010), stress (Wullschleger et al., 2009; Beniwal et al., 2010; Chen and Polle, 2010; Janz et al., 2010), seasonality (Brunner and Nilsson, 2004; Rohde et al., 2011; Larisch et al., 2012), interactions with other organisms including mycorrhizal fungi (Philippe and Bohlmann, 2007; Felten et al., 2009; Luo et al., 2009; Nehls et al., 2010; Schnitzler et al., 2010). *Populus* is a well-established model tree for biotechnology and molecular biology research due to the availability of the fully sequenced genome of *Populus trichocarpa* (Torr. and Gray) genotype “Nisqually-1” (Tuskan et al., 2006), the ease of *in vitro* cultivation (Kang and Chun, 1997; Kang et al., 2009) and the ease of vegetative propagation by cuttings, except aspen (Lubrano, 1992; Confalonieri et al., 2003).

Cloning of poplars *in vitro* enables the production of homogeneous plant material throughout the year (McCown, 1997; Kang et al., 2009). *In vitro* micropropagation is one of the most important multiplication techniques for different purposes like breeding, genetic and biotechnological research, and mass production of commercial clones (Nool et al., 2002; Altman, 2003; Confalonieri et al., 2003; Häggman et al., 2007; Yadav et al., 2009).

Most of the existing studies on poplar growth protocols focus on a single poplar species (Iordan-Costache et al., 1995; Phan et al., 2004; Kang et al., 2009; Peternel et al., 2009; Thakur et al., 2012). In the present paper we report differing techniques and growth conditions for different poplar species. We describe the micropropagation of the species *P*. *trichocarpa* (section Tacamahaca), *P*. *tremula* (section Populus), *P*. *euphratica* (section Turanga), and the hybrid *Populus × canescens* (syn. *P. tremula × P. alba*) under axenic conditions and the transfer of the plantlets into soil for outdoor experiments.

Further attention has been paid to the cultivation of poplar with ectomycorrhizal fungi because those interactions are a hallmark of forest trees, important for tree nutrition and stress tolerance (Nehls et al., 2010; Habib et al., 2013). Growth of the symbiotic partners together under axenic conditions is important for the elucidation of the underlying molecular processes. We, therefore, also describe growth conditions for *Laccaria bicolor* and *Paxillus involutus*, which are well-established ectomycorrhizal model systems for the establishment of the symbiotic interaction with poplar. The genome of *L. bicolor* is available and that of *P. involutus* is currently being sequenced (Martin et al., 2008; Marmeisse et al., 2013). Therefore, the combination of poplar with sequenced model fungi is especially useful to unravel the molecular ecology of tree-mycorrhizal interactions.

**Figure 1:**
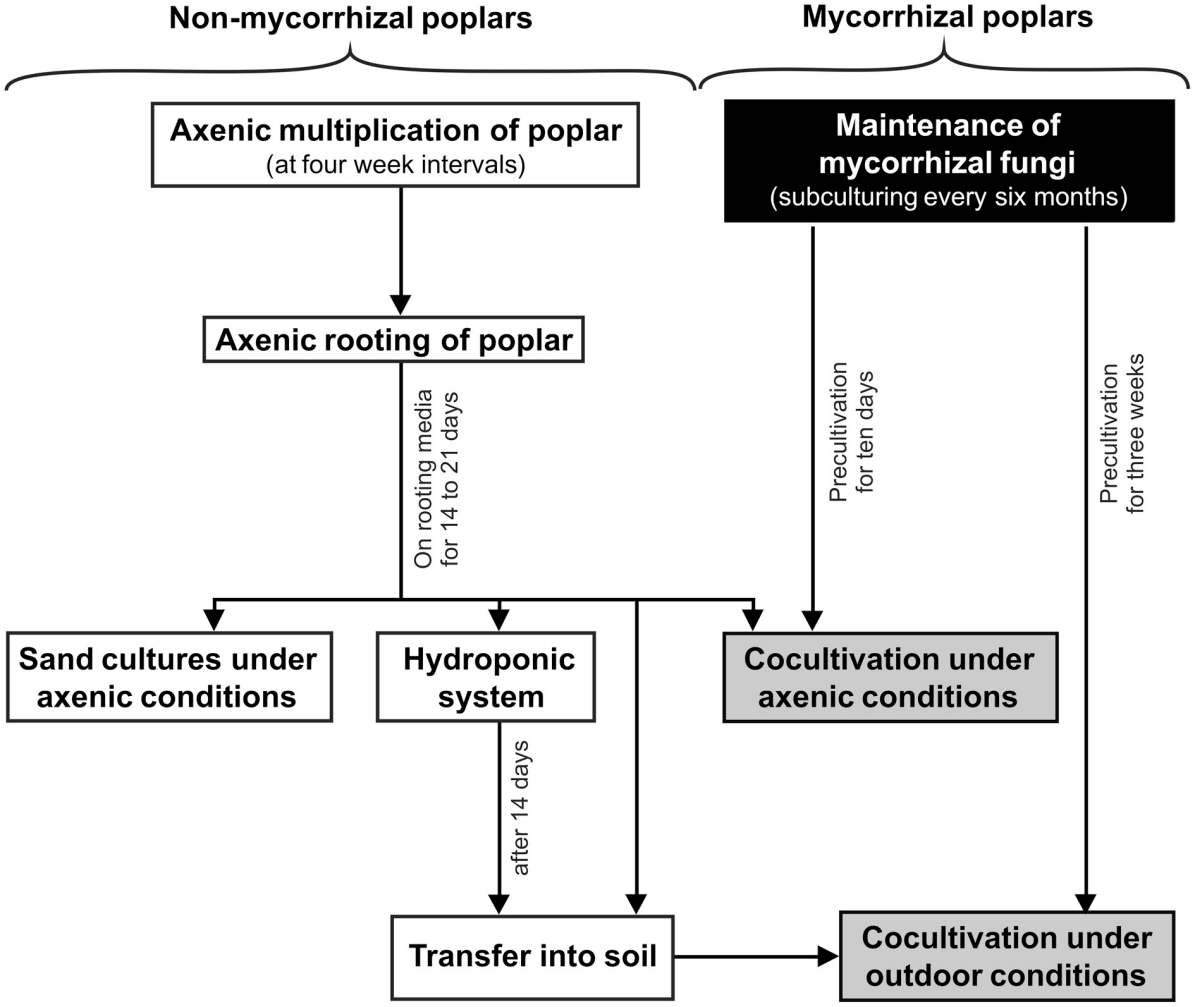
**Flow chart for growing poplars (*Populus sp*.) under mycorrhizal or non-mycorrhizal conditions**. The chart displays poplar growth conditions for axenic or outdoor cultivation (white color), for the cultivation of mycorrhizal fungi (black color), and the co-cultivation of mycorrhizal fungi and poplar (gray color). Approximated cultivation times are indicated.

The goal of this work is to describe well-established protocols, give practical advice and examples for the work with poplars and their ectomycorrhizal associates. The first step before micropropagation can start is the establishment of tissue cultures from field-grown trees. Protocols for shoot regeneration from tissue explants have been reported for a number of poplar species (Vinocur et al., 2000; Noël et al., 2002; Phan et al., 2004; Yadav et al., 2009; Maheshwari and Kovalchuk, 2011) and readers are referred to those studies. Here, we focus on the *in vitro* multiplication of different poplar species and their preparation for different experimental approaches under highly controlled axenic or outdoor conditions. To guide the reader through the different stages of poplar and fungal cultivation together or separately, we present a flow chart for the work schedule (Figure 1). The subheadings of the different protocols and propagation steps are repeated in the flow chart to facilitate cross-referencing.

## Axenic multiplication of poplar

For research purposes homogeneous plant material for the replication of the experiments is essential. For the micropropagation of *Populus* × *canescens*, *P*. *trichocarpa*, *P*. *tremula*, and *P*. *euphratica* media based on the original protocols of Murashige and Skoog (1962) (=MS medium) and Lloyd and McCown (1980) for woody plants (=WPM medium) were used. Suitable multiplication media for different poplar species are listed in Table 1 and the composition of the media has been compiled in Table 2.

**Table 1:** **Multiplication and rooting media for different poplar species**.

**Poplar species**	**Multiplication media**		**Rooting Media**	
	**MS**	**WPM**	**SH**	**WPM**
*P. × canescens*	X	–	X	X^*^
*P. trichocarpa*	–	X	–	X
*P. tremula*	X	–	X	–
*P. euphratica*	X	X	–	X

Abbreviations refer to MS, Murashig & Skoog, WPM, Woody Plant Medium, and SH, Schenk Hildebrandt media. Suitable medium are marked with an X.

* Rooting of P. × canescens on WPM medium is slower than on SH medium.

**Table 2:** **Composition of Murashig & Skoog (MS), Long Ashton (LA), Schenk Hildebrandt (SH), and Woody Plant Medium (WPM) for multiplication and rooting of poplar plantlets**.

**Compound**	**MS^$^ (g l^−1^)**	**LA (g l^−1^)**	**SH (g l^−1^)**	**WPM (g l^−1^)**	**Supplier^&^**
**MACRO-ELEMENTS**					
CaCl_2_ × 2H_2_O	0.44	–	0.2	–	Merck
CaCl_2_	–	–	–	72.5 × 10^−3^	Duchefa
Ca(NO_3_)_2_ × 4H_2_O	–	21.25 × 10^−2^	–	47.126 × 10^−2^	Merck
KH_2_PO_4_	0.17	81.64 × 10^−3^	–	0.17	Merck
KNO_3_	1.9	20.22 × 10^−3^	2.5	–	Merck
K_2_HPO_4_	–	72 × 10^−4^	–	–	Merck
K_2_SO_4_	–	–	–	0.99	Duchefa
MgSO_4_ × 7H_2_O	0.37	0.074	0.4	–	Merck
MgSO_4_	–	–	–	18.054 × 10^−2^	Duchefa
(NH_4_)H_2_PO_4_		–	0.3	–	Merck
NH_4_NO_3_	1.65	–	–	0.4	Duchefa
**MICRO-ELEMENTS**					
CuSO_4_ × 5H_2_O	2.5 × 10^−5^	32 × 10^−6^	25 × 10^−6^	25 × 10^−5^	Merck
FeNaEDTA	–	36.71 × 10^−4^	–	36.7 × 10^−3^	Sigma
H_3_BO_3_	62 × 10^−4^	6.18 × 10^−4^	0.003	6.2 × 10^−3^	Roth
MnSO_4_ x H_2_O	0.01	3.38 × 10^−4^	0.01	22.3 × 10^−3^	Merck
Na_2_MoO_4_ x 2H_2_O	2.5 × 10^−4^	16.92 × 10^−4^	25 × 10^−5^	25 × 10^−5^	Merck
ZnSO_4_ x 7H_2_O	86 × 10^−4^	5.76 × 10^−5^	0.003	86 × 10^−4^	Merck
KJ	83 × 10^−5^	–	75 × 10^−5^	–	Merck
CoCl_2_ x 6H_2_O	2.5 × 10^−5^	–	25 × 10^−6^	–	Merck
CoSO_4_ x 7H_2_O	–	1.12 × 10^−5^	25 × 10^−6^	–	Merck
**VITAMINS**					
Glycine	0.002	–	0.002	0.002	Duchefa
myo Inositol	0.1	–	0.1	0.1	Duchefa
Nicotin Acid	5 × 10^−4^	–	5 × 10^−4^	5 × 10^−4^	Merck
Pyridoxine HCl	5 × 10^−4^	–	5 × 10^−4^	5 × 10^−4^	Sigma
Thiamine HCl	1 × 10^−4^	–	1 × 10^−4^	1 × 10^−3^	Merck
C_10_H_12_FeN_2_NaO_8_	36.7 × 10^−3^	–	36.7 × 10^−3^	–	Sigma
Saccharose	20	–	25	–	Duchefa
Gelrite^§^	3	–	2.8	–	Duchefa

The concentrations are given as final concentrations.

§ Before Gelrite was added the pH was adjusted with NaOH or HCl to 5.7. The total volume was brought to 1 l with distilled H_2_O and the media were autoclaved at 121°C for 20 min at 2.2 bar.

$ For MS medium 25 mg benzyl amino purine (BAP, Sigma, Steinheim, Germany) was dissolved in 50 ml ethanol and filled up to 100 ml with bidest. From this stock solution 0.8 ml was used for 1 l medium.

& Suppliers: Carl Roth, Karlsruhe, Germany; Merck, Darmstadt, Germany; Duchefa, Haarlem, Netherlands; Sigma, Steinheim, Germany; Serva, Mannheim, Germany.

For micropropagation, we used glass jars (Schott, Jena, Germany) with a diameter of 14 cm and a height of 5 cm. The jars were covered with a glass Petri dish (Schott, Jena, Germany). After selection of the multiplication medium (Tables 1, 2), it was solidified with 3% Gelrite. MS medium was used as the basis medium for micropropagation. Its pH value was adjusted to 5.8 before sterilization by autoclaving (HST 6 × 6 × 6, Zirbus Technology GmbH, Bad Grund, Germany) at 121°C and 2.2 bar for 20 min. All procedures, which require sterile conditions, were carried out under laminar air flow (Typ S2020 1.8, Thermo Scientific, Schwerte, Germany). For multiplication of the various poplar species, we used approximately 1–2 cm long stem cuttings of ca. 4-week-old plantlets, each containing one leaf. About 10–15 cuttings were transferred vertically into one glass jar containing an about 2 cm thick layer of half-strength MS medium. The glass jars were then sealed with Parafilm™ (Bemis Flexible Packing, Neenah WI, USA) and incubated in a growth room under controlled environmental conditions [26°C, 60% relative air humidity, 16 h/8 h light/dark cycle with a photosynthetic active radiation (PAR) of 150 μmol photons m^−2^s^−1^ at plant height supplied by lamps (Osram 18W/640 white light, München Germany)]. After 4 weeks, the plantlets had a height of about 4–6 cm and 6–8 leaves. The plantlets were then used for further multiplication, by separating the shoot into 1–2 cm long cuttings each containing one leaf. The multiplication medium does not induce root formation and is, therefore, mainly used for the maintenance of stock cultures and production of high numbers of shoot cuttings under controlled conditions. All stock cultures were maintained in duplicates or triplicates.

One risk of micropropagation is the contamination of the cultures with microorganisms. Therefore, the glass jars were checked regularly for any kind of visible contamination. Jars with contaminated plantlets were immediately disposed. When transgenic plantlets had to be discarded, the media and plantlets were sterilized (121°C, 20 min, 2.2 bar).

For optimal performance of the plantlets in the subsequent experiments, the growth media for the poplar species (see Table 1), the right size of the plantlets and also the jar size has to be adapted. We are using glass jars of 7 cm height with 150 ml medium for the culture of *P. × canescens, P. euphratica*, and *P. tremula*. Because *P. trichocarpa* grows faster than the other poplar species glass jars of 12 cm height with 150 ml medium are recommended for the latter species. In case of suboptimal conditions, e.g., medium composition or space limitations, the plantlets can show symptoms of nutrient deficiencies or growth suppression during the experimental period (Poorter et al., 2012).

## Axenic rooting of poplar

To induce rooting of the plantlets, the unrooted freshly cut microcuttings were transferred to either Schenk Hildebrandt (SH medium; Schenk and Hildebrandt, 1972) or to WPM with hormones (Tables 1, 2). Because of the different growth characteristics of different poplar species, the cuttings rooted within 14–21 days (cf. Figure 1).

## Sand cultures under axenic conditions

Rooted poplars can also be grown in sand as the supporting medium under axenic conditions. The plants are fertilized by addition of suitable nutrient solutions. The advantage of sand is that it can be easily sterilized and supports the formation of more typical root systems than agar or Gelrite-based media. The plantlets can be removed easily for further analyzes. The nutrition can be controlled by addition of fertilizer solution because the sand contains no nutrients.

**Figure 2:**
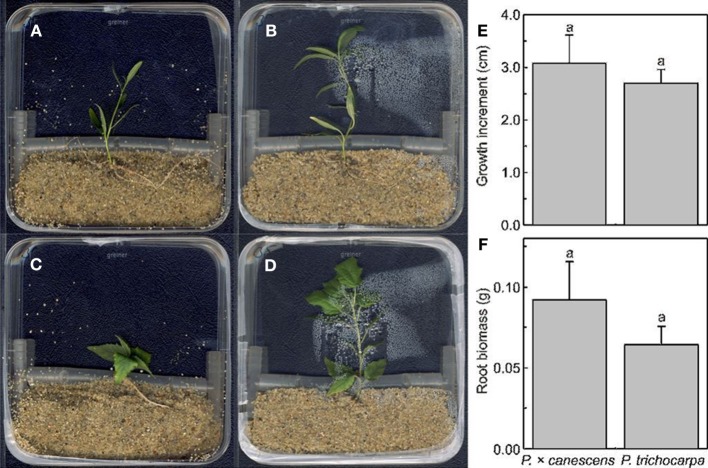
**Poplar plantlets in a Petri dish system under axenic conditions in sand**. Petri dishes with *P. trichocarpa* immediately after planting **(A)** and after 5 weeks **(B)**. Petri dishes with *P*. × *canescens* immediately after planting **(C)** and after 5 weeks **(D)**. Height increment **(E)** and fresh root biomass of *P. trichocarpa* and *Populus* × *canescens* after 5 weeks **(F)**. Data are means (±SE, *n* = 12). The same letters indicate the absence of significant difference with *p* < 0.05.

In our study we grew *P. × canescens* and *P. trichocarpa* in sand cultures. For this purpose square Petri dishes (12 × 12 cm, Carl Roth GmbH & CoKG, Karsruhe, Germany) were equipped with a self-made spacer, which consisted of an 11 cm long flexible tube (Rotilab PVC tube, Ø inside 6 mm, outside 10 mm, Carl Roth) connected with tube couplers (Carl Roth) on both ends for stabilization (Figures 2A–D). For the fixation of the plantlets, a small indentation was cut into the tube with a razor blade and the plantlet was inserted. The spacer was introduced at 4 cm from the bottom to fix the position of plants and soil. The lower part was filled with 75 g of autoclaved sand (Ø 0.71–1.25 mm particle size, Melo, Göttingen, Germany; Figure 2). We used plantlets that had been grown for 18 d on WPM medium (as described above). Plantlets with three to four roots with a length of 0.5–1 cm were transferred into the sand culture. *P. × canescens* or *P. trichocarpa* plantlets were carefully placed in the indentations of the spacer tube with the roots placed in the sand. An aliquot of 8 ml of the sterilized growing medium (WPM) was added to the sand. Afterwards the Petri dishes were sealed with Parafilm and kept in a growth room under the same environmental conditions as before (see above). To prevent light exposure of the roots, the bottom halves of the Petri dishes were wrapped in foil. The plates were arranged vertically in self-made plastic racks for optimal light exposure and grown under same environmental conditions as before. We kept the plants in these systems for 5 weeks without further fertilizer addition (Figure 2). Growth was measured weekly by scanning the Petri dishes with a resolution of 300 dpi (CanScan 4400F, Canon Inc, Tokyo, Japan) and image analysis software (DatInf® Measure 2.1, Tübingen, Germany). After 5 weeks of growth, the plantlets were harvested.

The cultivation period is limited by the size of the Petri dishes and nutrient availability. Figures 2A,C show typical plantlets after insertion into the system and after 5 weeks when they had grown about 3 cm in height (Figures 2B,D). We did not observe significant differences in height or root biomass between *P*. × *canescens* and *P. trichocarpa* (Figures 2E,F).

## Hydroponic systems

Rooted poplars can easily be grown in hydro-cultures after acclimation. In this case water is the supporting medium. A water-based culture has the advantage that it allows the access to the roots for e.g., physiological or biomass measurements during the experimental treatment (Poorter et al., 2012) and that the nutrient supply can be controlled.

**Figure 3:**
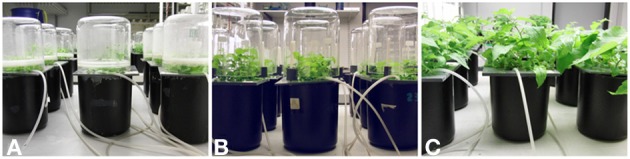
**Hardening of *Populus* × *canescens* plantlets in a hydroponic system**. The plants are shown after 1 week **(A)**, 2 weeks **(B)**, and 3 weeks **(C)** in hydroculture.

We cultivated poplar plants in aerated hydroponic solutions in black covered or painted plastic containers filled with Long Ashton (LA) nutrient solution (Hewitt and Smith, 1975) (Table 2). Aeration was achieved by bubbling air, which was supplied by an in-house connection through the nutrient solution after filtering through quartz wool filters (HEKAtech GmBH, Wegberg, Germany). The *in vitro* pre-cultured rooted plantlets were transferred into the LA solution after 3 weeks on rooting media. The containers were covered with a plastic lid into which holes had been drilled to insert the plants. The plants were fixed with small sponges in the perforated lid. The nutrient solution was renewed once a week or more frequently when the plants were very large. The plants were grown in the growth room as before (photoperiod of 16 h light and 8 h darkness and 150 μmol quanta m^−2^s^−1^ PAR at 26°C and 60% air humidity). After removal from the rooting medium, the plantlets are extremely sensitive to desiccation. It is, therefore, necessary to acclimatize the plantlets to ambient conditions. For this purpose, the plants were covered by a glass beaker, which was gradually lifted during the course of 14 days (Figures 3A–C). We used hydroponically grown poplars to conduct salt stress experiments (Brinker et al., 2010; Janz et al., 2012) or to investigate the effects of heavy metals (Elobeid et al., 2011). We conducted our hydroponic experiments mainly in controlled growth rooms, but have also applied the system successfully under greenhouse conditions (e.g., Elobeid and Polle, 2012).

## Transfer into soil

*In vitro* micropropagated poplar plantlets are not adapted to ambient outdoor conditions. They are acclimatized to high air humidity and a low UV irradiation. The leaves have a thin cuticle and a poor regulation of transpiration (Pospóšilová et al., 1999). Therefore, the transfer of poplars from *in vitro* to *ex vitro* conditions is a critical step, with a high risk of plant mortality. To prevent the plants from wilting, high relative air humidity has to be maintained during hardening. For hardening of poplar plantlets, it is possible to use hydroponic systems as described above. After 2 weeks the plants can be transferred into soil.

Another possibility is the direct transfer of micropropagated plants from the *in vitro* culture into soil or sand. Here, it is also important to keep the plants in high air humidity, which can be achieved by covering the plants with a transparent plastic bag for up to 2 weeks. The bag is gradually lifted. We potted the poplars in soil (Fruhstorfer Erde Type N, Hawite Gruppe GmBH, Vechta, Germany) or sand/peat mixtures [two parts of peat (REWE, Köln, Germany), eight parts coarse sand (Ø 0.71–1.25 mm; Melo, Göttingen, Germany) and two parts fine sand (Ø 0.4–0.8 mm; Melo, Göttingen, Germany)]. Growth in sand/peat requires regular fertilization. Cultivation in a sand/peat mixture is useful, if roots are to be analyzed or if controlled nutrient supply is important. Regular watering is important, especially for plants in sand/peat mixtures.

The plants can be grown in pots in acclimatized rooms, greenhouses, or outdoors. Recording of the environmental variables (light intensities, day/night lengths, air humidity, and temperatures) is necessary to control the experimental conditions. In the greenhouse, additional illumination may be required to achieve day lengths >14 h when the plants are grown in seasons with short days. The size of the pot depends on the duration of the experiment.

**Figure 4:**
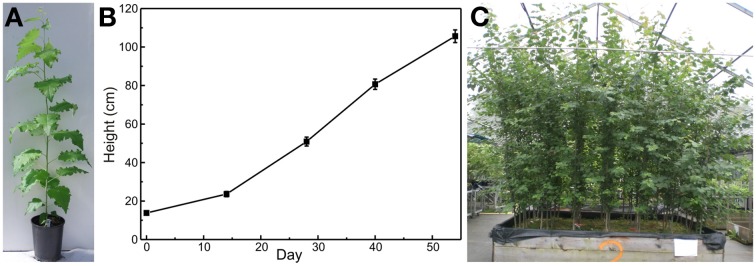
***Populus* × *canescens* plant grown outdoors (A), height development during the growth phase of 54 days (B), and long-term growth in soil filled boxes (C)**. The potted poplars were grown in 3l pots from the day of planting to day 54 (mean ± SE, *n* = 16).

In our studies *Populus* × *canescens* was grown for 2 months in 3l pots in Frühstorfer Erde Typ N under outdoor conditions (Figure 4). The potted plants were gradually acclimated to outdoor conditions by gradually increasing the exposure times outdoors and initially avoiding full sun. After 54 days, the plants reached the height of about 1 m (Figure 4), an aboveground biomass of 14 g and a belowground biomass of 4 g. If the plants are intended to be grown for longer time periods, pots with larger volumes, or use of large boxes are recommended (Figure 4). Selecting the correct pot size is a trade-off between a higher number of replicates (small pots) and a longer duration of the study (large pots or boxes). In the large boxes, we maintained poplars for up to 2 years and heights up to 2.5–3 m (Figure 4). With a governmental permission, growth of transgenic poplars under outdoor conditions is possible (e.g., Behnke et al., 2010, 2012). Field trials with transgenic poplars have also been reported (Walter et al., 2010; Danielsen et al., 2012). However, because of the strict regulation in many countries, such field trials are not common (Strauss et al., 2009).

## Maintenance of mycorrhizal fungi

For research on poplar—ectomycorrhizal fungal (EMF) interactions, the plant and the fungus have to be pregrown in parallel. Therefore, the maintenance of pure fungal cultures is essential. It enables a replication of experiments and the use of the same fungal isolates for further studies. Over the last decades, the techniques and methods for preservation of fungal isolates have been improved. We describe suitable media, growth conditions, and cultivation periods for *Laccaria bicolor* and *Paxillus involutus*.

Media useful for fungal stock cultures are, e.g., Melin–Norkrans medium and the Pachlewski medium. We used a modified Melin–Norkrans medium (MMN) or the Pachlewski media P05 for the maintenance of ectomycorrhizal fungi (after Marx, 1969; Deveau et al., 2007). The compositions of the media are listed in Table 3.

**Table 3:** **Composition of the modified Melin–Norkrans medium (MMN), the Pachlewski medium (P05), and the sugar-reduced Pachlewski medium (P20)**.

**Compound**	**MMN (g l^−1^)**	**MMN_low_(g l^−1^)**	**P05 (g l^−1^)**	**P20 (g l^−1^)**	**Supplier^&^**
Glucose D+	10	2	20	1	Roth
Maltose D+	3	–	5	–	Merck
Di-NH_4+_ tartrate	–	2.5	0.5	0.5	Merck
KH_2_PO_4_	0.5	0.5	1	1	Merck
(NH_4_)_2_SO_4_	0.25	0.25	–	–	Merck
MgSO_4_ × 7H_2_0	0.15	0.15	0.5	0.5	Duchefa
CaCl_2_ × 2H_2_O	0.05	0.05	–	–	Merck
NaCl	0.025	0.025	–	–	Merck
FeCl_3_^b^	0.01	0.01	–	–	Merck
Thiamine HCl^b^	0.01 × 10^−2^	0.01 × 10^−2^	0.01 × 10^−2^	0.01 × 10^−2^	Merck
B^b^	–	–	2.23 × 10^−4^	2.23 × 10^−4^	Yara^a^
Cu^b^	–	–	3 × 10^−5^	3 × 10^−5^	Yara^a^
Fe EDTA^b^	–	–	6 × 10^−4^	6 × 10^−4^	Yara^a^
Mn^b^	–	–	6.4 × 10^−4^	6.4 × 10^−4^	Yara^a^
Mo^b^	–	–	2.7 × 10^−5^	2.7 × 10^−5^	Yara^a^
Zn^b^	–	–	2.12 × 10^−4^	2.12 × 10^−4^	Yara^a^
Agar	10	10	20	12	Duchefa

The concentrations are given as final concentrations.

Before Agar was added the pH of the media was adjusted with NaOH or HCl. The MMN medium was adjusted to 5.2 and the pH of the P05 and P20 medium was adjusted to 5.8. The total volume of the media was brought to 1 L with distilled H_2_O before sterilization by autoclaving at 121°C for 20 min at 2.2 bar.

& Suppliers: Carl Roth, Karlsruhe, Germany; Merck, Darmstadt, Germany; Duchefa, Haarlem, Netherlands; Yara Nanterre Cedex, France.

a The microelement solution Kanieltra from Yara, which contained B, Cu, Fe EDTA, Mn, Mo, and Zn, was used in a stock solution of 10% (v/v).

b Stock solutions of 1% (w/v) FeCl_3_, 0.1% (w/v) thiamine HCl, and 10% (v/v) Kanieltra microelement solution were sterile filtered through a 0.2 μm filter unit (Filtropur from Sarstedt, Nümbrecht, Germany) using single-use syringes (Omnifix from B. Braun Melsungen AG, Melsungen, Germany) under a laminar air flow unit and stored at 4°C. Of each of the stock solutions (FeCl_3_, thiamine HCl, and Kanieltra) 1 ml l^−1^ were added, when the temperature of the autoclaved media was ~60°C.

For maintenance of stock cultures Petri dishes with a diameter of 10 cm were filled with 30 ml medium and inoculated with a single fungal plug (diameter = 1 cm) in the center. The plates were sealed with Parafilm and kept under controlled-environmental growth conditions at 23°C in permanent darkness to produce materials for experiments. For storage, cultures were kept at 4°C in permanent darkness on MMN media and subcultured onto the MMN media every 6 months.

One risk of frequent subculturing, especially while working with different fungal isolates, is the chance of contamination of the cultures. Therefore, meticulous attention has to be paid to sterility. The identity of the fungal species should be confirmed regularly by molecular analysis of the internal transcribed spacer sequence (ITS) as described elsewhere (Lang et al., 2011). Saprotrophic long-term cultures of ectomycorrhizal fungi may result in the loss of the ability of the EMF to form functional symbiosis. Therefore, most EMF must occasionally be employed to form ectomycorrhizas with a suitable host and then re-isolated from the roots. However, to our experience this is not required for *Paxillus involutus* (strain MAJ), which we used the last 15 years (Brandes et al., 1998), neither for *Laccaria bicolor* (strain S238N Maire P.D. Orton provided by F. Martin, INRA, Nancy, France), which has been successfully used for about 20 years (Henrion et al., 1994). Petri dish systems with synthetic media can be used to investigate the performance of the ectomycorrhizal fungi in absence of their hosts. For example, Müller et al. (2013) revealed profound differences in the volatile profiles of EMF including *P. involutus* and *L. bicolor*, which may play roles in plant-fungal communication.

## Cocultivation under axenic conditions

Culture systems for the cultivation of poplars with *L. bicolor* or *P. involutus* under *in vitro* conditions have been described, e.g., by Felten et al. (2009) and Gafur et al. (2004). We successfully used a modified co-culture system after Felten et al. (2009), in which *L. bicolor* was pregrown on a cellophane membrane (Deti GmbH, Meckesheim, Germany) on top of Pachlewski medium P20 (Table 3). Advantages of using cellophane on top of a media are the supply of the fungus with all necessary nutrients, while at the same time preventing its growth into the medium. This allows the fungus to be easily transferred from one Petri dish to a new Petri dish or its use for analyses.

**Figure 5:**
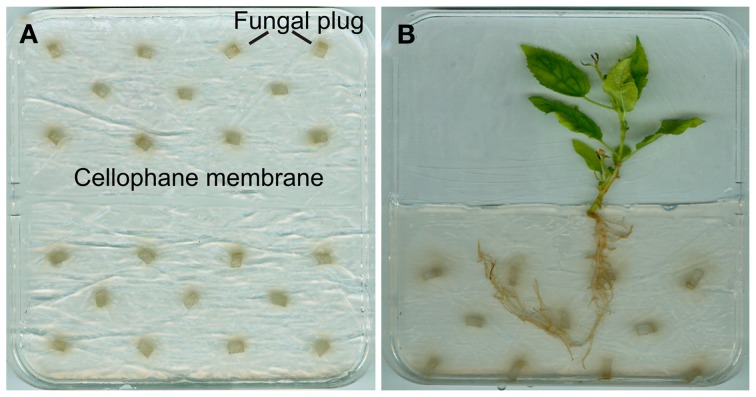
**Cocultivation of *Populus* × *canescens* with *L. bicolor* under axenic conditions. (A)** Preculture of *L. bicolor* in a Petri dish on a cellophane membrane on P20 medium. It is possible to equip this system with two membranes in one Petri dish. **(B)** Cocultivation of *P.* × *canescens* with *L. bicolor*.

Square Petri dishes (12 × 12 cm) were filled with 50 ml sugar-reduced P20 medium. Cellophane membranes were cut into halves (6 × 12 cm). The membranes were first boiled twice for 30 min in distilled water and subsequently autoclaved twice. Two membranes were placed in one Petri dish onto the P20 medium next to each other (Figure 5A). Eleven fungal plugs of *Laccaria bicolor* cultures, which had been pre-cultured on P05 media, were placed on the membrane (Figure 5A). The Petri dishes were sealed with Parafilm and kept at 23°C in permanent darkness. The regular placement of the fungal plugs on the cellophane membranes ensured the regular coverage of the cellophane surface with actively growing fungal mycelium after 10 days of culture. The arrangement on the cellophane membrane as well as the precultivation time should be adjusted according to the growth of the fungus. It is therefore recommended to analyze the growth of the fungus on the used media before starting the final experiment.

For inoculation with *L. bicolor*, *P. × canescens* was micropropagated *in vitro* and grown on rooting media in glass jars for 3 weeks (as described above). For the cocultivation square Petri dishes (12 × 12 cm) containing 50 ml of sugar-reduced P20 medium were used. After solidification, the upper half of the medium in the Petri dish was removed. The remaining medium was covered by a cellophane membrane (6 × 12 cm) with or without 10 days old fungal mycelium of *L. bicolor*, pregrown on P20 media. A 3-week old, rooted poplar plantlet was placed on the fungal mycelium (Figure 5B). The shoot and leaves of the plant were located in the empty half of the Petri dish. The Petri dishes were sealed with Parafilm. To prevent exposure of the roots and the fungal mycelium to light, the lower halves of the Petri dishes were wrapped in aluminium foil. The plates were arranged vertically in racks and kept in a growth room at 26°C with a day/night cycle of 16 h/8 h. In general, the cultivation period of poplars in this system is limited by the size of the Petri dish, the size of the plantlets and the nutrient availability. In our experiments, poplar plantlets did not show symptoms of nutrient deficiencies or shortage of space during the 21 days of their growth.

To grow slightly larger mycorrhizal plants under axenic conditions, another co-culture system was used for the mycorrhization of *Populus* × *canescens* with the ectomycorrhizal fungus, *Paxillus involutus*. The fungal strains *P. involutus* NAU and MAJ were maintained on MMN media. For precultivation of the fungi, Petri dishes (diameter: 15 cm) were filled with 15 ml of a modified low carbohydrate MMN media (MMN_low_; Table 3). A sterilized cellophane membrane (diameter: 7 cm) was placed on the agar and a mycelium plug of either *P. involutus* MAJ or NAU was placed on to the cellophane membrane. The poplar plantlets were pregrown for 3 weeks in glass jars, which contained 160 ml MMN media.

**Figure 6:**
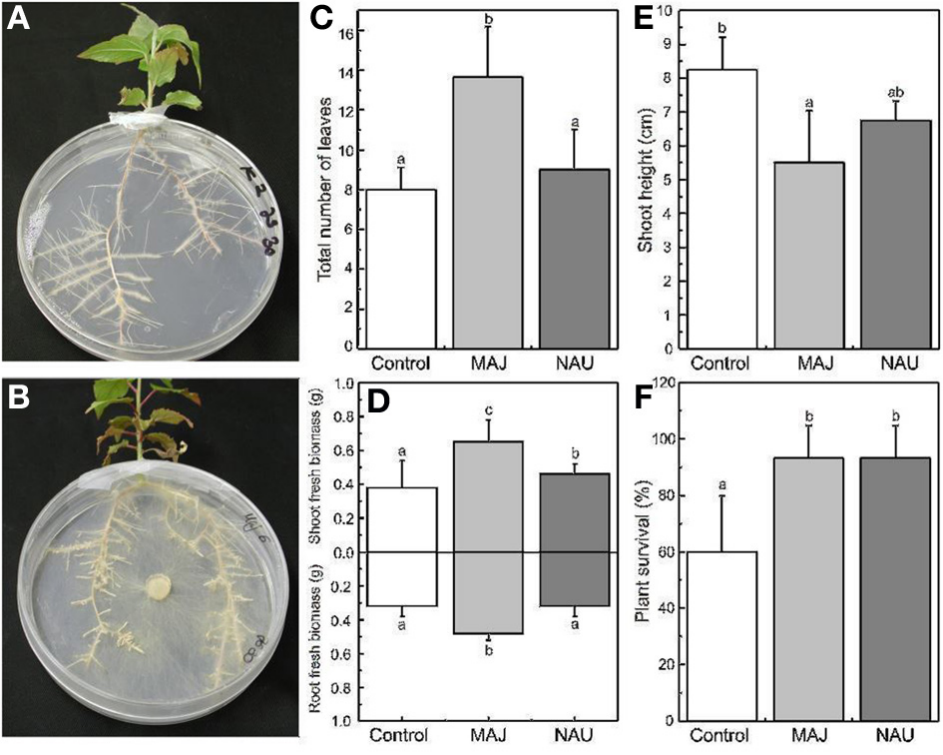
**Petri dish system for the cocultivation of *Populus* × *canescens* with *P. involutus* under axenic conditions**. All data are shown for plantlets 4 weeks after the transfer into the Petri dishes. **(A)** Control, non-mycorrhizal plantlet, and **(B)** mycorrhizal plantlet. **(C)** Total leaf number, **(D)** shoot and root biomass, and **(E)** shoot height of plantlets inoculated with *P. involutus* strain MAJ or strain NAU (mean ± SD, *n* = 5). **(F)** Survival of non-inoculated control plantlets and plants inoculated with *P. involutus* strain MAJ or strain NAU (mean ± SD, *n* = 15). Different letters indicate significant differences with *p* < 0.05.

To assemble the plant-Petri dish system, a hole (8 mm) was made in the sidewall of the Petri dish using a hot forceps. The shoot of about 3-week-old poplar plantlets was placed outside the Petri dish, whereas the roots were spread either on non-inoculated medium (Figure 6A) or on the 2-week-old fungal mycelium (Figure 6B). The Petri dishes were closed with Parafilm and wrapped in aluminum foil. They were placed in special planting chambers, which were covered with a translucent plastic top. The system was maintained under high humidity of 70–75%, a temperature of 22–23°C and 200 μmol m^−2^s^−1^PAR in an acclimatized growth room. Especially, at the beginning the plants were very sensitive to desiccation and wilted easily. Furthermore, the system is more prone to infections by other microbes than the system in which the plants are grown completely inside the Petri dish and therefore has to be controlled regularly to discard contaminated dishes.

**Figure 7:**
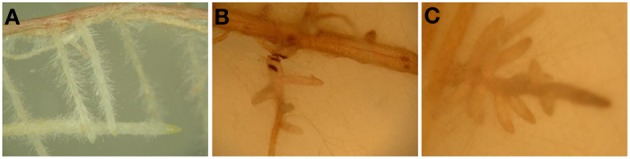
**Mycorrhizal and non-mycorrhizal roots of *P. × canescens***. All pictures were taken 4 weeks after transfer into the Petri dishes. **(A)** Root of a control plant with dense root hairs, **(B)** Changes in root tip morphology induced by *Paxillus involutus* strain NAU with a weak mycelium and brownish spots, and **(C)** typical dichotomously branched root tips with dense mycelium of *P. involutus* strain MAJ.

We cultured poplars and ectomycorrhizal fungi in this system for up to 4 weeks (Figures 6A,B). Figure 6B shows hyphae extending from the fungal plug to the roots. Poplars inoculated with *P. involutus* strain MAJ, which forms a functional mycorrhiza, developed more leaves, taller shoots, and more biomass than poplars without ectomycorrhiza (Figures 6C–E). In contrast to the strain MAJ, the strain NAU is not able to form functional ectomycorrhizas (Gafur et al., 2004), but nevertheless stimulated biomass production (Figure 6D) and increased the survival rate of the plantlets (Figure 6F). A closer inspection of the roots showed very dense formation of root hairs for the non-mycorrhizal plants (Figure 7A), coverage with a thin yellowish mycelium of the roots of the NAU inoculated plants (Figure 7B) and the characteristic extramatrical mycelium around new root tips and typical dichotomously branched roots for the MAJ inoculated plants (Figure 7C).

Controlled poplar-ectomycorrhizal systems are most useful to investigate the molecular basis of plant-fungal interactions and to analyze the biochemical composition of fungus, plants, and their combination without confounding effects of interfering organisms (e.g., Felten et al., 2009; Reich et al., 2009).

## Cocultivation under outdoor conditions

To investigate non-mycorrhizal and mycorrhizal poplars under greenhouse or outdoor conditions, we employed different inoculation techniques: (a) cocultivation of the plant with the fungus *in vitro* and transfer of the mycorrhizal plants into soil. This results in high mycorrhization rates, but is very time consuming; (b) direct transfer of micropropagated *Populus × canescens* plants into soil, which had been mixed with homogenized, liquid cultures of the ectomycorrhizal fungus as described by Langenfeld-Heyser et al. (2007). Non-inoculated control plants have to be planted into soil which had been mixed with culture medium. It is also possible to pre-cultivate the plants in hydroponic solution. This intermediate step is more time-consuming, but generally leads to stronger plants; (c) direct transfer of micropropagated *Populus × canescens* plants into soil together with solid fungal inoculum. Because the protocols (a) and (b) have already been described in a detailed manner (Gafur et al., 2004; Langenfeld-Heyser et al., 2007; Beniwal et al., 2010), we describe here protocol (c).

**Figure 8:**
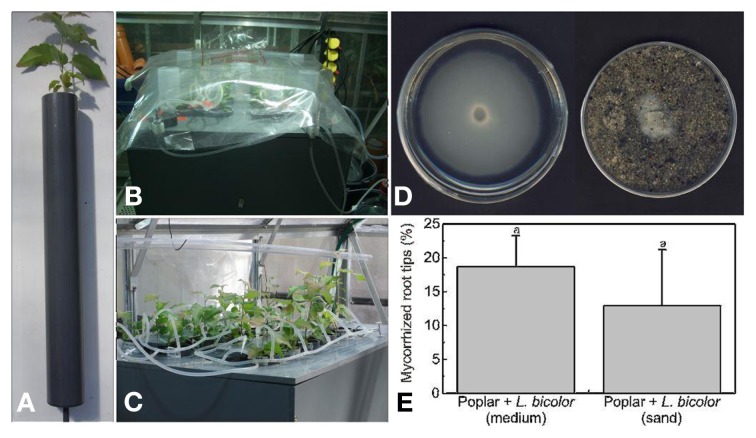
**Cocultivation of *P. × canescens* with *L. bicolor* in growth tubes**. *P.* × *canescens* plants in growth tubes with a sand/peat mixture **(A)**, plants in the acclimatization phase covered with a transparent plastic bag **(B)**, removal of the bags after 2 weeks **(C)**, pre-grown *L. bicolor* in Petri dishes on agar media or sand **(D)**, percentage of mycorrhizal root tips of *P. × canescens* grown in a sand/peat mixture inoculated with *L. bicolor* pregrown on agar medium or sand **(E)** (mean ± SE, *n* = 5). The same letters indicate the absence of significant difference with *p* < 0.05.

*L. bicolor* was cultivated in Petri dishes (diameter: 10 cm) on the solid media MMN (Table 2) for 3 weeks at 23°C in darkness (Figure 8D). One fungal plug was placed in the center of the medium. For control plants, Petri dishes with media but without fungus were prepared. After 3 weeks the total content of one Petri dish with or without fungal mycelium was cut into small pieces using a scalpel and mixed into 660 ml of a sand/peat mixture. The sand/peat mixture has been described above.

In another approach the fungus was pre-grown on sand, which has the advantage that agar or Gelrite media are not transferred along with the fungus into the co-culture system. For this purpose a sandwich medium was prepared. The bottom of a Petri dish (diameter: 10 cm) was filled with 30 ml modified MMN media (Table 2). A cellophane membrane that had been boiled twice in distilled water (30 min) and autoclaved twice was placed on top of solidified medium. Then 70 g autoclaved sand was filled on the medium and a plug of vigorously growing mycelium was placed in the center on the sand (Figure 8D). Two ml autoclaved liquid MMN medium (diluted 1:1) was added. The plates were incubated at 23°C in permanent darkness. After 3 weeks the fungal mycelium had grown throughout the sand, but the cellophane membrane prevented direct growth into the solidified medium. For controls, sand without the addition of fungal plugs was prepared in the same way. The sand of one Petri dish with or without *L. bicolor* was mixed with 660 ml of the sand/peat mixture [two parts peat (REWE, Köln, Germany), eight parts coarse sand (Ø 0.71–1.25 mm Melo, Göttingen, Germany) and two parts fine sand (Ø 0.4–0.8 mm Melo, Göttingen).].

*P.* × *canescens* plantlets were rooted as described above and either planted directly into soil or after 2 weeks of hydroponic growth in LA solution. Prior to use the sand/peat mixture was mixed with the pieces of the solid medium or the sand, in which *L. bicolor* had been grown for 3 weeks. Six hundred and sixty milliliters sand/peat mixture with or without *L. bicolor* was filled in one growth tube (diameter = 5 cm, length = 41 cm; Figure 8A). The plants were planted into the growth tubes and immediately irrigated. The growth tubes were placed vertically in a specially designed PVC box (Figure 8B). To protect the plants against evaporation they were initially covered with transparent plastic bags (Figure 8B). The plastic bags were gradually lifted during the course of 2 weeks (Figure 8C). The plants were automatically irrigated with 10 ml of LA solution three times daily. For irrigation, a 5 L bottle (Schott, Jena, Germany) was filled with the nutrient solution and set under an air pressure of 50 kPA by an electronic pump (N022 AN1.8 from KNF Neuberger, Freiburg, Germany). The bottle was connected by an irrigation system of silicon tubes (Carl Roth, Karlsruhe, Germany) with each plant. The irrigation system was comprised of silicon tubes, T-pieces (Carl Roth, Karlsruhe, Germany), a magnetic valve (Bürkert, Ingelfingen, Germany) and 200 μl pipette tips (Sarstedt, Nümbrecht, Germany), which were placed in the growth tubes and served as nozzles. The irrigation was controlled with an integrated magnetic valve. The magnetic valve was connected to an electronic timer (Grässlin, Georgen, Germany) and opened every 8 h for 20 s when the bottle was under pressure. Excess solution could flow off through a hole at the bottom of the growth tubes. The plants were grown under controlled environment conditions with a 16 h light and 8 h darkness rhythm. After 21 days, about 13–19% of the roots were mycorrhized with *L. bicolor* (Figure 8E). These mycorrhization rates were similar to those observed for axenic poplars on agar or gelrite plates (Figure 8E). After longer incubation times, the colonization rate of the root tips was further increased. This and similar culture systems have been successfully applied to study the influence of the EMF on plant stress tolerance and nutrient uptake (Brandes et al., 1998; Luo et al., 2009; Li et al., 2012).

## Conclusions and outlook

Growing poplars for research requires the careful selection of suitable growth conditions for various poplar species. In this method paper, reliable and well-established techniques for experimental setups with poplar under axenic and outdoor conditions have been described and illustrated by examples. To synthetize ectomycorrhizal poplars, the plant, and the fungus have to be pregrown in parallel, which has to be planned carefully. An overview of cultivation options and growing periods for research on poplars with and without mycorrhiza is summarized in the flow chart (Figure 1). Co-culture of poplars with ectomycorrhizal fungi promoted plant growth and vitality *in vivo* and *in vitro*. After extended co-cultivation mycorrhization rates in the range of 50–60% of the root tips can be found (e.g., Luo et al., 2009). Similarly, the colonization of field grown poplars is initially low, but increases to almost 100% in second year after planting (Danielsen et al., 2013). Therefore, the conditions reported here result in realistic EMF colonization rates.

We also reported the *in vitro* propagation of different poplars species which are particularly useful for specific research areas. *P*. × *canescens* is widely used because well-established transformation protocols exist for this hybrid for a long time (Leplé et al., 1992). *P. euphratica* is now the best investigated model tree to study salt tolerance in woody species (Chen and Polle, 2010). *P. trichocarpa* is a riparian species of high commercial value because it can be used for the production of fast-growing hybrids. The Eurasian aspen, *P. tremula* occurs on marginal soils and in forests, and therefore, is currently of increasing interest to study adaption to drought and nutrient stress (Kleemann et al., 2011; Euring et al., 2012). With exception of aspen, the other poplar species can easily be multiplied by woody cuttings. However, for a homogeneous quality of the plant material, the use of micro-propagated material is strongly recommended. Furthermore, plants for experiments can be produced at any time, whereas the best time to obtain woody cuttings is late winter.

### Conflict of interest statement

The authors declare that the research was conducted in the absence of any commercial or financial relationships that could be construed as a potential conflict of interest.
